# Outcomes of Secondary Prevention among Coronary Heart Disease Patients in a High-Risk Region in Finland

**DOI:** 10.3390/ijerph15040724

**Published:** 2018-04-11

**Authors:** Teppo Repo, Markku Tykkyläinen, Juha Mustonen, Tuomas T. Rissanen, Matti Ketonen, Maija Toivakka, Tiina Laatikainen

**Affiliations:** 1 Department of Geographical and Historical Studies, University of Eastern Finland, 80101 Joensuu, Finland; markku.tykkylainen@uef.fi (M.Ty.); maija.toivakka@uef.fi (M.To.); 2 North Karelia Hospital District, 80210 Joensuu, Finland; juha.mustonen@siunsote.fi (J.M.); tuomas.rissanen@siunsote.fi (T.T.R.); matti.ketonen@siunsote.fi (M.K.); tiina.laatikainen@siunsote.fi (T.L.); 3 National Institute for Health and Welfare (THL), 00271 Helsinki, Finland; 4 Institute of Public Health and Clinical Nutrition, University of Eastern Finland, 70211 Kuopio, Finland

**Keywords:** secondary prevention, electronic medical records, coronary heart disease, primary care, quality of care, rural health, risk factors of CHD, geospatial health, health-care planning

## Abstract

Despite comprehensive national treatment guidelines, goals for secondary prevention of coronary heart disease (CHD) have not been sufficiently met everywhere in Finland. We investigated the recorded risk factor rates of CHD and their spatial differences in North Karelia Hospital District, which has a very high cardiovascular burden, in order to form a general view of the state of secondary prevention in a high-risk region. Appropriate disease codes of CHD-diagnoses and coding for percutaneous coronary intervention (PCI) and coronary artery bypass grafting (CABG) were used to identify from the electronic patient records the patient group eligible for secondary prevention. The cumulative incidence rate of new patients (*n* = 2556) during 2011–2014 varied from 1.9% to 3.5% between municipalities. The success in secondary prevention of CHD was assessed using achievement of treatment targets as defined in national guidelines. Health centres are administrated by municipalities whereupon the main reporting units were municipalities, together with composed classification of patients by age, gender and dwelling location. Health disparities between municipalities, settlement types and patient groups were found and are interpreted. Moreover, spatial high-risk and low-risk clusters of acute CHD were detected. The proportion of patients achieving the treatment targets of low-density lipoprotein cholesterol (LDL-C) varied from 21% to 38% between municipalities. Variation was also observed in the follow-up of patients; e.g., the rate of follow-up measurements of LDL-C in municipalities varied from 72% to 86%. Spatial variation in patients’ sociodemographic and neighbourhood characteristics and morbidity burden partly explain the differences in outcomes, but there are also very likely differences in the care process between municipalities which requires a study in its own right.

## 1. Introduction

The scale and severity of the cardiovascular disease epidemic makes prevention and good management of this disease one of the linchpins of public health. Coronary heart disease (CHD) in particular constitutes a growing medical and economic burden on societies. Secondary prevention after acute coronary syndromes, by intervening in adverse lifestyles and intensifying prescriptions of anti-hypertensive and lipid-lowering drugs, is a crucial step in the prevention of recurrent events [1].

The association of cardiovascular disease and deprivation of neighbourhood is relatively well studied [2,3], as is for example the importance of geographic accessibility to acute interventional cardiology services [4], but there exist very few extensive studies on the outcomes of secondary prevention of CHD combined with the geospatial variation in the follow-ups. Those that do exist indicate a number of challenges in the effective deployment of secondary prevention strategies and often disregard detailed spatial aspects.

EUROASPIRE surveys have shown that success in secondary prevention of cardiovascular disease (CVD) varies between European countries. Across Europe, there are variations in the management of risk factors and preventive medication between countries, socio-economic groups and genders [1,5,6]. Studies have shown that risk factor prevention goals of blood pressure and low-density lipoprotein cholesterol (LDL-C) levels set by the European Society of Cardiology in their ESC Guidelines in clinical practice and Joint European Societies (JES) guidelines [1,5] have not been achieved by the large majority of CHD patients in Europe.

Kaufmann et al. [7] analysed the attainment of LDL-C goals of CHD patients at Kaiser Permanente Colorado, US, and showed that a considerable proportion of high-risk patients are able to attain the LDL-C goal of 1.8 mmol/L when specific attempts to achieve this goal are made. Nevertheless, over 50% of patients did not attain the treatment target, and demographic and medical characteristics were found to affect the attainment of the LDL-C goal [7]. A registry-based Taiwanese study assessed LDL-C management in the context of the secondary prevention of CVD and showed development in LDL-C management, but also indicated a need for better implementation of the national guidelines in clinical practice [8]. Furthermore, in the US, a study by Jones, Nair and Thakker, showed that a need for improvement in medication adherence among high-risk patients was found in lipid goal attainment among high-risk patients (CHD and CHD-risk equivalent) [9].

Challenges in risk factor management have been identified. For example, the British Heart Foundation has reported that cardiac rehabilitation programmes reduced an already small proportion of smokers from 7% to 5%, but that the same programmes were not as effective in reducing BMI across the United Kingdom [10]. A study by De Smedt et al. indicated differences in risk factor clustering between genders, wherein risk factor management for older women with low socio-economic status (SES) was found to be the most suboptimal [11]. Novel, better targeted approaches for improving risk management are therefore needed.

Despite the challenges faced by secondary prevention programmes, a few studies have pointed to encouraging results from these programmes. A systematic review and meta-analysis by Murphy et al. concluded that organizational secondary prevention programmes reduced the risk of cardiac-related deaths by 26%, and suggested that targeting the programmes at high-risk patients would be beneficial [12], while Hawkes et al. argue that telephone-delivered CHD secondary prevention programmes can improve patients’ engagement in lifestyle modification [13].

The success of secondary prevention of CHD depends on the management of risk factors such as high blood pressure, smoking, diabetes, low high-density lipoprotein cholesterol (HDL-C) and elevated LDL-C. Previously, it was laborious to gather reliable data on patients with CHD at a regional level in Finland, but research on outpatient care is now easier in our study since the incorporation of the districts’ several local patient records into a single data system. Implementation of more exhaustive and geocodable patient databases means that outcomes of secondary prevention in outpatient care can be analysed more accurately and in different spatial scales along with new approaches [14]. Thus far, however, such analyses have been scarce.

In Finland, primary health care is run by the municipality and specialised care by a collaboration between several municipalities in the hospital district. Areas inside the hospital district’s service region vary in terms of rural/urban status and economic structure, which is reflected in the variation in demographics, socio-economic characteristics and living standards among municipalities. Earlier studies have shown that rural areas and small towns outside the commuting zone of a regional centre suffer most from low employment and demographic vulnerabilities, such as demographic ageing, gender imbalance and outmigration, which is also reflected in the health disparities [15,16]. The proportion of the CHD risk group of elderly males is largest in remote rural areas where the population decline and ageing is most rapid due to selective outmigration and low birth rates [17].

The objective of this study was to analyse the CHD incidence rates and management of CHD risk factors in order to evaluate the state of secondary prevention of CHD in a peripheral Nordic high-risk region exemplified by the North Karelia Hospital District. To achieve this objective the study detected areas with elevated risk for acute CHD incidences and assessed factors affecting the spatial variation in incidence rates and treatment outcomes. A practical goal was to identify area types and patient groups that are in need of attention, which will be useful for health-care planning in similar rural regions.

## 2. Materials and Methods

### 2.1. Study Population

**Figure 1:**
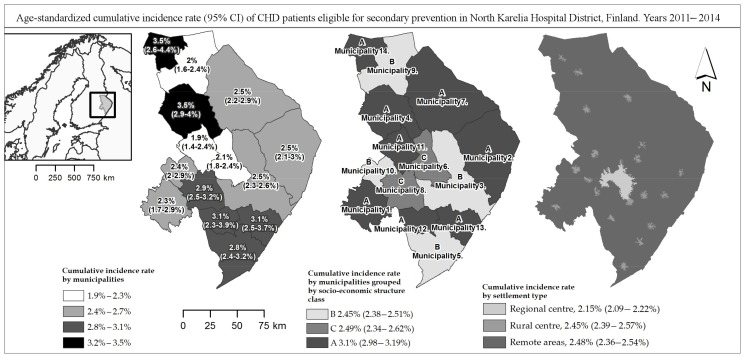
Age-adjusted four year cumulative incidence rate of acute coronary heart disease and/or invasive treatment in North Karelia Hospital District in 2011–2014 classified by municipalities and settlement types. (CI = confidence interval, CHD = coronary heart disease). Municipalities’ socio-economic structure classification: A = Sparsely populated, low education, unemployment, primary production, high demographic vulnerability; B = Moderately dense population, high education, service; C = High education, high employment, diverse industry, low demographic vulnerability.

Patient data were obtained from electronic patient records of North Karelia Hospital District. These records cover both primary and specialized care for all 14 municipalities in the service region, with 169,000 inhabitants (Figure 1). This joint database of patient records has been in commission since 2010. Laboratory results are comparable to each other since all samples in the hospital district are analysed in one central laboratory.

The patient group consisted of individuals with diagnosed acute coronary syndrome (ACS) and/or patients who had undergone a preventive cardiac operation, i.e., percutaneous coronary intervention (PCI) or coronary artery bypass grafting (CABG) in 2011–2014. Patients eligible for CHD secondary prevention were identified from patient records using national operative codes of CABG and PCI and 10th revision of the International Statistical Classification of Diseases and Related Health Problems coding (ICD-10) for unstable angina and myocardial infarction (ACS) I20.0 (incl. sub-codes) and I21 (incl. sub-codes). Code I20 without sub-classifications was excluded from the selection criteria in order to minimize the number of uncertain diagnoses. Patients aged 35–84 years who were alive at the end of the year 2014 were included. Individuals with the above-mentioned diagnosis or those who had PCI or CABG before 2010 were excluded in order to form a more homogeneous patient group representing new CHD cases. Place of domicile was used to allocate patients to municipalities and to three different settlement types, in analysing the spatial differences and to create thematic map visualizations, as well as placing the patients’ geolocations in grid cells for geospatial analysis of disease clustering. The total number of patients selected after exclusions was 2556, 64.4% of whom were men. The data include the dwelling location, gender, date of birth, laboratory results of blood lipids, measured body mass index (BMI), blood pressure, recorded smoking status and diagnoses of other chronic diseases.

### 2.2. Ethical Approval

The ethics approval for the study was obtained from the ethics committee of the Kuopio University Hospital on 13 November 2012. The review of the committee adheres to the Medical Research Act (No. 488/1999).

### 2.3. Data Analysis

To assess the risk of random variation by municipality, the cumulative incidence rate of CHD patients diagnosed with ACS and/or having had PCI/CABG from 2011–2014 was calculated for each municipality with a 95% confidence level. Due to demographic differences, incidence rates were age-standardized using demographics data from Statistics Finland. In addition, data from Statistics Finland were used in classifying the municipalities by socio-economic factors into three municipality types.

Using choropleth maps for visualization of incidence rates at a fine spatial scale in North Karelia Hospital District may cause outlier and anonymity problems due to the sparse population in remote geographical areas. To overcome this, the spatial scan statistic procedure was applied to analyse the disease clustering in the region. Input data encompass the number of patients and the size of the corresponding aged population in 5 km × 5 km grid cells. In addition, the proportion of over 65 year old population in grid cells was calculated to be used as a covariate in age-adjusted spatial scan statistics analyses. The matching population data in grid format from the year 2015 was obtained from the Spatial Structure and Urban Form (YKR) data set, which mostly consists of coordinated based statistical data from Statistics Finland, and is managed by the Finnish Environmental Institute. The selected method, grid size of input data and cluster scales were applied to complement the results of the incidence rates, and the parameters were selected to maintain the anonymity of the patients’ dwelling locations even in very sparsely populated areas.

The spatial scan statistics was run with SaTScan 9.4 software (developed by Martin Kulldorff together with Information Management Services Inc., Boston, MA, USA) [18] using Poisson probability model with circular scanning windows. This method is known for being sensitive for selected parameters such as scale sizes [19]. To take this into account, the analyses were run with several different scales to detect clusters. Clusters with a maximum 8% and 30% of population at risk were selected for the comparison of the impact of the scale effect in the final analyses.

Processes and outcomes of secondary prevention were evaluated using the rate of patients’ follow-up activity and by comparing treatment outcomes to the recommendations of Finland’s Current Care Guidelines [20]. Only recordings and measurements taken from at least one month after an acute cardiac event or invasive treatment were included. Earlier recordings of BMI and smoking status taken immediately before or at the time of diagnosis/procedure were also included in cases when the person was registered of normal weight or a non-smoker.

The treatment target for LDL-cholesterol in CHD patients, based on the national guidelines for dyslipidaemia, is <1.8 mmol/L [21]. A higher, less strict cut-off of 2.5 mmol/L was also used in our analysis for the sake of comparability to earlier research. Outcomes of LDL-cholesterol treatment were considered as the main indicator of achieving the sufficient level of the secondary prevention of CHD. This indicator was selected due to its relatively active measurement rate and the importance of it as a risk factor.

Based on national guidelines, the cut-off value of 1.7 mmol/L was used for triglycerides, while for HDL-cholesterol 1.0 mmol/L was used for men and 1.2 mmol/L for women [21]. The cut-off values of 25.0 kg/m^2^ (overweight) and 30.0 kg/m^2^ (obesity) were used for the body mass index (BMI). A blood pressure cut-off level of 140/90 mmHg was used in the analysis, following national guidelines [22]. Smoking status was recoded as a binomial variable from two categorical variables in patient records. Both daily and occasional smokers were treated as smokers in our analysis. In addition, comorbid conditions were taken into account to analyse the differences between the two patient profiles. Morbidity burden was defined by categorizing patients with or without other chronic disease diagnosis. Age was categorized in three approximately evenly sized groups: 35–64, 65–74 and 75–84 years old. The geocoded patient’s dwelling locations were classified into three different settlement types where they each have roughly the same number of patients. The regional centre covers the city of the regional capital and its commuting zone within a road distance of 20 km, rural centres cover the areas with a 5 km road distance area around small municipal centres and non-urban population centres with a public health-care unit. The third area group, remote areas, was formed from the remaining land area and mostly consists of sparsely populated remote rural areas (Figure 1).

A patient grouping with 18 categories was compiled from these age, gender and geographic location variables (Supplementary Table S1). Moreover, municipalities were categorized using factor analysis into three socio-economic structure classes (A, B and C) by the sociodemographic characteristics and their economic structure (Supplementary Table S2, Figure 1).

## 3. Results

### 3.1. Cumulative Incidence of CHD with ACS and/or Invasive Treatment

**Figure 2:**
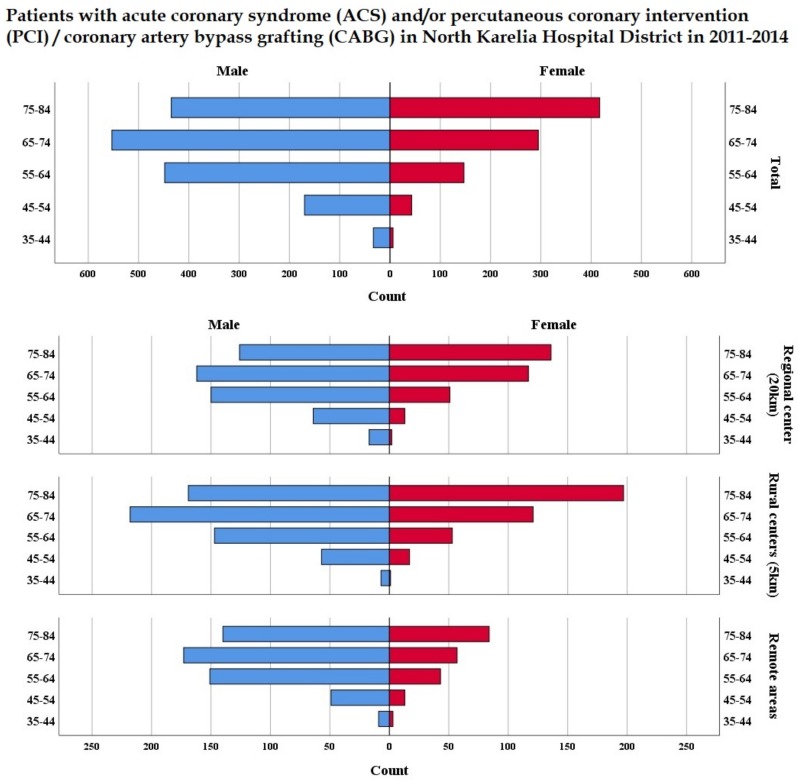
Age structure of patients with acute coronary syndrome (ACS) and/or percutaneous coronary intervention (PCI)/coronary artery bypass grafting (CABG) in North Karelia Hospital District catchment area in 2011–2014 classified by settlement types.

CHD cases were most prevalent among males in the age group of 65–74 whereas the number of female patients increases with age relatively evenly (Figure 2). Only 27.7% of patients in remote areas were females (Supplementary Table S1). The proportion of CHD patients who were eligible for secondary prevention was found to vary by municipality and by settlement types. Age-adjusted cumulative incidence rate during the study period was substantially higher among males in all municipalities, varying from 2.4% to 4.8% (Supplementary Figure S1), whereas the incidence rate of ACS or PCI/CABG in females varied from 1.0% to 2.4% (Supplementary Figure S1) and the total incidence rate for both genders varied from 1.9% to 3.5% by municipality (Figure 1).

Generally, the highest incidence rates were found in sparsely populated primary production-dominated municipalities, strained by low education level and high levels of unemployment and demographic vulnerabilities (Class A. 3.4% crude, 3.1% adjusted). More densely populated municipalities with relatively high education levels and a large proportion of service sector economy had lower incidence rates (Class B. Crude and adjusted rate somewhat below 2.5%). Mildly lower incidence rates were found in commuter towns adjacent to the regional centre (Class C. Crude 2% and adjusted 2.5%) (Figure 1).

When the region is classified by the settlement types the disparities of cumulative incidence rate of ACS or PCI/CABG between the regional centre, the commuting zone included, and the rest of the hospital district are clear. The lowest rate was in the regional centre (1.9% crude/2.2% age-adjusted) and highest in rural centres (2.9% crude/2.5% age-adjusted). The incidence rate in the class of remote rural areas was similar as found in rural centres, but with somewhat lower crude rate (2.5% and 2.5% respectively) (Figure 1). A relatively low incidence rate in remote areas among females was found (1.49% crude, 1.60% adjusted).

With the spatial scan statistics method, it is possible to detect high- and low-risk areas of diseases with different spatial scales [23]. The disease clusters, created with SaTScan software, revealed the opposing areas where relative risks for acute coronary incidences or having PCI or CABG are higher and lower than expected.

**Figure 3:**
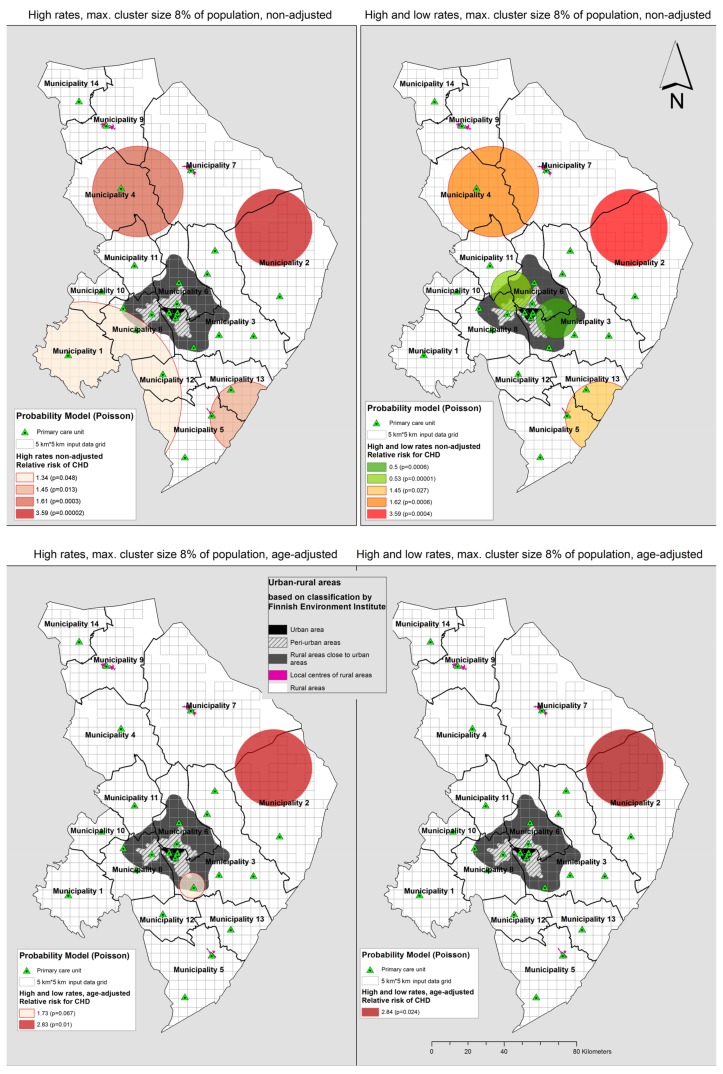
Disease clusters and low risk clusters of acute coronary heart disease and/or invasive treatment in 2011–2014 analysed at cluster size of maximum 8% of population at risk in North Karelia Hospital District.

**Figure 4:**
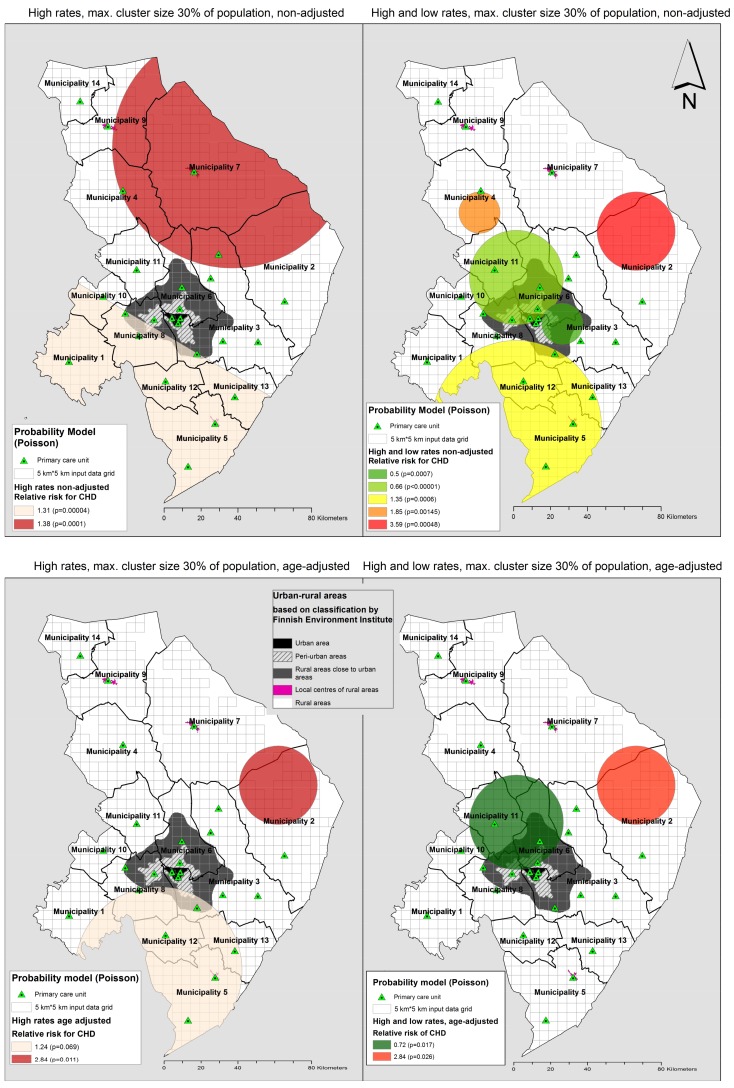
Disease clusters and low risk clusters of acute coronary heart disease and/or invasive treatment in 2011–2014 analysed at cluster size of maximum 30% of population at risk in North Karelia Hospital District.

The highest relative risk (RR) cluster (RR = 3.59) was detected from unadjusted analyses at both 8% and 30% (at 30% scale, only at a simultaneous high- and low-risk analysis). This very sparsely populated (0.27 inhabitants per km^2^) cluster with an approx. 1400 km^2^ surface area is located between municipality 2 and 7 in the eastern part of the hospital district, near the Russian border. The same cluster was detected in age-adjusted analysis (RR = 2.84) at all scales (Figure 3 and Figure 4).

At the 30% scale, the four high-risk unadjusted clusters detected at the 8% scale were merged into two larger clusters. The bigger cluster includes the entire municipality 7 along with large areas around it covering a surface area of 7500 km^2^. The other high-risk area covers 5760 km^2^ at a scale of 30%, including the entire southern part of the hospital district (Figure 4).

The latter cluster was also detected in an age-adjusted analysis at 30% (RR = 1.24) (Figure 4). A geographically small high-risk cluster (RR = 1.73) near the regional centre was detected in the age-adjusted analysis at an 8% scale, but without statistical significance (*p* = 0.067) (Figure 3).

In a simultaneous high- and low-rates scan, the low-risk clusters were found from the areas near the regional centre (RR = 0.5–0.66 unadjusted, RR = 0.72 age-adjusted). The high-risk areas of simultaneous scan for both high- and low-risk clusters differed from the only high-rates scan, as expected. The unadjusted analysis of both high and low clusters at an 8% scale resulted in one high-risk cluster less, and at a 30% scale smaller sized high-risk clusters were detected around the low-risk areas (Figure 3 and Figure 4).

### 3.2. Recording Activity

Measurement and recording activity of CHD risk factors varied greatly among municipalities and with gender and age. In particular, follow-up of blood pressure diverged within classifications.

LDL-C was measured for 77% of patients (Supplementary Table S2). LDL-C recording activity was more successful among males in all age and spatial groups, except for rural centres and remote areas in the 65–75 age group. (Supplementary Table S1). Variation in recording activity of LDL-C was moderate between municipalities (72.5–86.7%, SD = 4.0%) (Supplementary Table S2) as well as between patients grouped by age, gender and settlement type (66.9–86%, SD = 4.2%) (Supplementary Table S1), but some disparities were present. The lowest LDL-C follow-up rate of females was in the regional centre, regardless of the age (Supplementary Table S1).

Blood pressure measured by a health professional was recorded for 56% of the patients (Supplementary Table S2). Recording was most active among 74–84-year-old patients in all settlement types (57–74%), except among males in rural centre (Supplementary Table S1).

**Figure 5:**
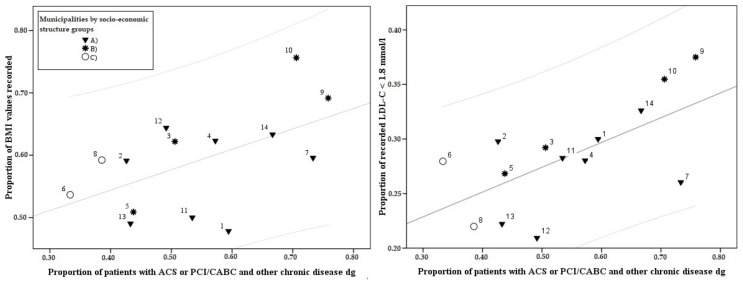
The municipality level associations between the proportion of multimorbid individuals and body mass index (BMI) follow-up rates and LDL-C management rates among patients with ACS and/or PCI/CABG in North Karelia Hospital District in 2011–2014. (BMI = body mass index, LDL-C = low-density lipoprotein cholesterol, ACS = acute coronary syndrome, PCI = percutaneous coronary intervention, CABG = coronary artery bypass grafting). A = Sparsely populated, low education, unemployment, primary production, high demographic vulnerability; B = Moderately dense population, high education, service; C = High education, high employment, diverse industry, low demographic vulnerability.

Approximately 60% of patients’ BMI values had been recorded into the patient database (Supplementary Table S1). BMI recording activity decreased by age from 65% (35–64 years old) to 56% (75–84 years old) and, for example, only about 51% of the age group of 75–84-year-old females in remote areas had their BMI recorded (Supplementary Table S1). Variation of BMI follow-up rate among the 18 age, gender and settlement type groups (51–72%, SD = 5.5%) was smaller than it was between municipalities (*n* = 14) (48–76%, SD = 8.1%). The BMI follow-up rate between municipalities associated positively with the proportion of CHD patients with comorbid conditions (Pearson correlation 0.547, *p* = 0.043) (Figure 5). Multimorbid females had a follow-up rate over 12 percentage points higher compared with patients having only ACS or PCI/CABG (Table 1).

Smoking status was recorded for 60% of patients (Supplementary Table S2). Recording activity was more constant among gender, age and settlement type groups (55–69%, SD = 4.2%) (Supplementary Table S2), than it was between municipalities (51–79%, SD = 7.3%) (Supplementary Table S1). The lowest recording of smoking status rate was in municipality class C (56%) in higher socio-economic conditions. The highest recording rate was in municipality class B (61%), where one municipality reached the 79% follow-up rate (Supplementary Table S2). These municipal differences are most likely to be explained by different diligence in incorporating lifestyle data in patient records in health-care centres during follow-ups.

### 3.3. Attainment of Treatment Targets

**Figure 6:**
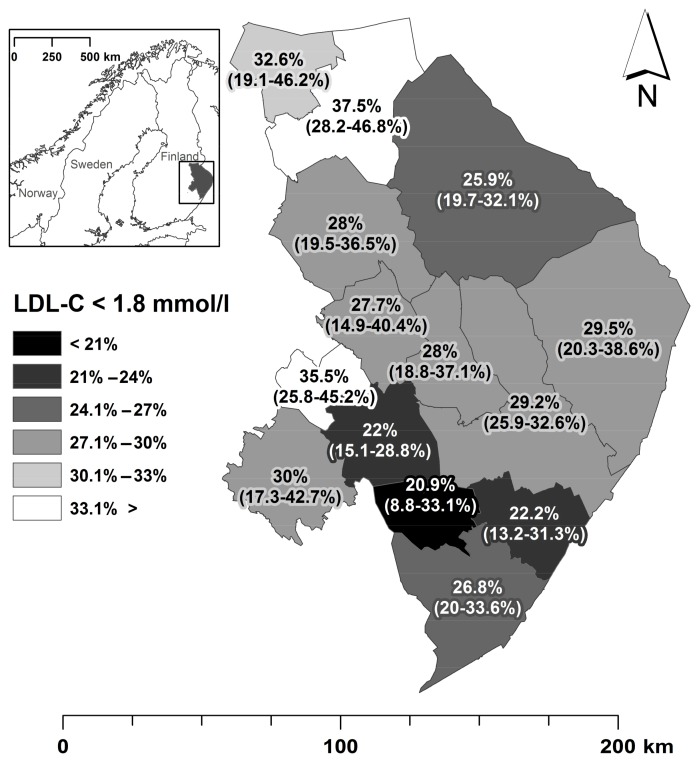
Low-density lipoprotein cholesterol (LDL-C) treatment target attainment among patients with ACS or PCI/CABC in North Karelia Hospital District in 2011–2014. (ACS = acute coronary syndrome, PCI = percutaneous coronary intervention, CABC = coronary artery bypass grafting).

Only 28% of selected patients were able to reach the national Current Care Guideline target for LDL-C level (<1.8 mmol/L). Our less strict target (<2.5 mmol/L), however, rendered 68% of patients able to reach the target level (Supplementary Table S2). The proportion of patients who achieved the target level of LDL-C (<1.8 mmol/L) varied from 21% to 38% (SD = 4.8%) between municipalities (Figure 6, Supplementary Table S1). High rates of suboptimal LDL-C levels in certain rural municipalities were due to commonly occurring high LDL-C records (>2.5 mmol/L) among female patients (Supplementary Table S1). The best attainment of LDL-C target level was achieved among 65–74-year-old male patients (38%) in remote areas and the worst was among 35–64-year-old females (20.4%) in the regional centre (Supplementary Table S1). Attainment of treatment targets (<1.8 mmol/L) was more successful in municipalities with high proportions of patients with comorbid conditions (Pearson correlation 0.642, *p* = 0.0133) (Figure 5). Overall, the attainment in treatment targets varied only somewhat less between municipalities (Supplementary Table S2) (SD = 4.8%) than between the age, settlement type and gender classification (SD = 5.0%) (Supplementary Table S1).

Almost a quarter of recorded HDL-cholesterol values were below the target levels (>1.0 for males, and >1.2 for females) [21]. Approximately 79% of followed-up patients were able to attain the target level for triglycerides (<1.7 mmol/L) (Supplementary Table S3).

The proportion of patients with high blood pressure (≥140/90 mmHg) was 53% (Supplementary Table S2). A notably high proportion (75.7%) of 65–74-year-old female patients living in remote areas had elevated blood pressure (Supplementary Table S1).

The prevalence of overweight and obese individuals was high among the studied patients. Almost 72% of males had BMI over 25 and 32% were obese. Sixty-eight per cent of females were overweight or obese (Supplementary Table S2) and in remote rural areas, the proportion was over 84% in age group of 65–74 years old (Supplementary Table S1).

Almost 12% of patients continued smoking after ACS or an invasive procedure. In all age and settlement types except for 35–64 year-old patients in regional centre and for 75–84 year-old patients in remote areas, tobacco use was more common among males. Overall smoking was approximately two times more common among male patients compared with females and notably more common in younger age groups in all settlement types among both genders (Supplementary Table S1).

## 4. Discussion

### 4.1. Summary of Findings

Age-standardized cumulative incidence rates of acute events or invasive procedures were found to vary between municipalities and settlement types, and the areas of high-risk disease clusters were detected. Significant variations in the incidence rates of ACS or invasive procedures and in the prevalence and follow-up activity of the risk factors of CHD were found between municipalities. Approximations of the disparities were further elaborated by comparing the outcomes by age, gender and place of domicile, and thus several patient groups with suboptimal treatment outcomes could be identified.

### 4.2. Incidence Rates

A division in age-adjusted cumulative incidence rates of ACS or an invasive procedure between different area types was clear, with some exceptions (Figure 1). In general, the more natural resource-based-oriented the economy of the area is, the higher is the proportion of the population under the risk of incidence of ACS and invasive procedures.

The highest age-adjusted incidence rates (3.1%) were found in deprived rural municipalities (in municipality socio-economic structure class A) (Figure 1). However, inside this class the municipality level rates varied from 1.9% to 3.5%. A moderate variation was also found in the municipalities of class B (2–2.8%), which covers areas with relatively high levels of education and a reasonably high proportion of service sector industries. This set of municipalities consists of the regional centre with its growing adjacent municipality and three town-like municipalities in the west, north and south. Class C consists of two rural commuter towns (2.1% and 2.9%) located near the regional centre and are characterized by high employment and diverse types of industry (Figure 1).

In the settlement type classification, the highest incidence rates were found in rural centres, with close proximity (<5 km) to primary care units, which in part roughly indicates that a better spatial accessibility to care does not improve primary prevention of CHD (Figure 1). However, it is worth noticing that a low proportion of new cases among women in remote areas suggests that persons at high risk from chronic conditions, especially women, have moved out from remote areas and most likely settled in rural centres.

Even though classifying the region in only three settlement types smooths out the geographical variation of cumulative incidence rate, the difference between the regional centre and the rest of the hospital district was clear, 2.15% and 2.48% respectively. However, an examination of rates at the municipality level reveals that a rural municipality number 11 near the regional centre with a relatively low sociodemographic status had the lowest age-adjusted cumulative incidence rates. Tentatively this could be explained by high rates of pre-hospital deaths among the persons in the lowest SES groups, reported in Finland earlier [24].

Spatial scan statistics were used to detect the clustering of high- and low-risk areas of new CHD incidences. The analyses highlighted the polarization of areas between low-risk commuter zones around the regional centre and high-risk remote areas. Most of the high-risk clusters were detected from rural areas of declining and ageing population, thus resulting in smaller and fewer clusters after age adjusting. However, some high-risk area results, as shown in thematic maps, for example in municipality 14, were not statistically significant in spatial scan statistics at any scale tested. The analysis revealed that there are municipalities in which both partial high- and low-risk clusters can be found (e.g., in municipalities 6 and 11, Figure 3). However, again, some of the high-risk clusters detected by spatial scan statistics covered too large an area to have practical use for health-care planning.

A very high-risk cluster covering a remote area over the borders of municipalities 2 and 7 was detected on several different scales, which was not shown in the choropleth incidence maps at municipal level (Figure 1 and Figure 3). Even when the very sparse and possibly sociodemographically unfavourable population distribution in the detected high-risk cluster in the eastern part of the region is taken into consideration, this finding indicates the need for further research into the cause of the elevated risk in this hot spot (Figure 3).

Nonetheless, the results should be taken only as the observed probabilities of the disease risk clustering in the area, taking into account, for example, the sensitivity of the method to the chosen parameters and the large variability of demographics, population density and other characteristics in the areas.

### 4.3. Recording Activity

The recording activity of CHD risk factors is more dependent on local public health treatment practices than it is on patient’s age, gender and place of domicile as the frequency of blood pressure, body mass index and smoking status data entries varied more between municipalities than between classification composed by age, gender and settlement type. Variation of LDL-C follow-up activity was somewhat smaller between municipalities than between the settlement types. However, disparities between municipalities in follow-up and recording practices are clear. This encumbers the interpreting of the attainment of treatment targets.

Overall, the higher rates of diagnosis and data entry may indicate generally a more thorough preventive care in the municipality. In addition, it may be possible that patients with more critical conditions are more likely be treated in public health care with specialized services and therefore the low follow-up rates in some geographical areas and age groups could indicate the higher use of private and occupational care instead of regular public care. Our results also show that patients with multimorbidity were followed up more effectively, which supports the conclusion that the gravity of illness impacts on the level of measuring intensity. Lower SES is associated with more frequent primary care visits, whereas people with high income may be more likely to use private health care, which is not included in our data [25]. However, in our study region, the availability of private services is low and chronic patients in Finland are typically treated in pubic health care.

### 4.4. Risk Factors/Attainment of Treatment Targets

In our study, the proportions of coronary patients (age 35–84) with LDL-cholesterol levels over 1.8 mmol/L (Supplementary Table S1) were somewhat smaller than the European averages in the EUROASPIRE IV survey (male 79%, female 84%, age <80 years), which also included CHD patients with acute myocardial infarction (ICD-10 I21) and acute myocardial ischaemia (ICD-10 I20) or PCI/CABG [1]. Less optimal treatment outcomes than ours were also reported by a Canadian study with a relatively similar patient group: with a 2.5 mmol/L target, the attainment rate was 52% [26], compared with 68% in our study. However, in the health-care system of Kaiser Permanente Colorado, US, the proportions of patients with a history of myocardial infarction or PCI/CABG and LDL-cholesterol over 1.8 mmol/L were lower [7]. When patients were classified by gender, age and settlement type, the variation of LDL-C attainment (20–38%, SD = 5%) (Supplementary Table S1) was similar to the total variation between municipalities (21–36%, SD = 4.8%) (Supplementary Table S2) indicating that treatment outcomes in LDL-C management may be more dependent on processes of care than the sociodemographic characteristics of patient population. Frequent suboptimal LDL-C scores were recorded, for example in the regional centre among females in the working age of 35–64 years. Only 20% had attained the treatment target while 45% exceeded the 2.5 mmol/L. Even though this age/gender group of patients was small (*n* = 66), this is in line with earlier studies showing worse management of risk factors among women and especially younger women [11,27,28].

Mean triglyceride levels in the study area were lower (1.36 mmol/L) than those found in Canada (1.75 mmol/L) [26] and in Taiwan among cardiovascular patients (1.58 mmol/L) [8] (Supplementary Table S3). The proportion of CHD patients without elevated triglyceride levels was 79%, which was higher than the results the EUROASPIRE III study reported for Finland (71%), and significantly higher than the European average (65%) in the same study [29].

The proportion of patients with high blood pressure was found to be considerably higher for both genders (50% males, 58% females) as compared with the EUROASPIRE IV study (42% and 44%, respectively) [1]. In the Canadian study, the percentage of CHD patients not achieving the blood pressure target of <140/90 mmHg (including diabetic patients with <130/80 mmHg) was 43% [26], and the percentage of CVD patients with suboptimal blood pressure in a Danish study (patients with ischaemic heart diseases, and cerebral and peripheral vascular diseases) was 62% [30]. Management of hypertension has in general been challenging in Finland and only about one-third of hypertensive patients achieve the treatment goals [31]. Elevated blood pressure in the 65–74 patient age group was notably high in remote areas among females (76%) and respectively in the regional centre among males (58%) (Supplementary Table S1).

Being overweight or obese was prevalent among CHD patients in our study (70%) (Supplementary Table S2). The percentage of overweight patients was even higher than this (males 83% and females 80%) in the EUROASPIRE IV study, in which the percentages of obese patients were 36% among males and 44% among females, compared with 32% and 35%, respectively, in our study [1]. The median BMI values among CHD patients in Canada and the US were 29 [26] and 31 [7], respectively, both significantly higher than in our study (27.6). The average BMI among Taiwanese CVD patients was 26 [8], which is significantly lower than in our study (Supplementary Table S3).

According to the Finnish Regional Health and Well-being Study (ATH), about 15% of the adult population in North Karelia are current smokers [32], indicating relatively poor smoking cessation rates among patients in our study, of whom 12% were still smoking after ACS or an invasive procedure. This result is similar to the proportion of smokers in the Canadian study (over 12%) [26]. The EUROASPIRE IV study reported 16% of patients (age < 80) as smoking currently [1]. A similar proportion (approx. 14%) of patients were current smokers in Taiwan [8]. In a study by Prugger et al., approximately half of the patients in the EUROASPIRE III study with ACS or PCI/CABC had quit smoking after hospitalization; however, one-quarter of the patients had no intention to give up smoking [33]. In our study, the large variations between municipalities (6.1–20%, SD = 3.8%) (Supplementary Table S2) and between age and gender groups by settlement types (1–30%, SD = 10.6%) were found in smoking status. Among male patients, smoking was most common in rural centres (30.2%) and among females, in the regional centre (28.9%), in the 35–64 age group (Supplementary Table S1), which is in line with observed gender, age group and SES differences in smoking in earlier studies [34].

### 4.5. Multimorbidity

The proportion of individuals with diagnosed comorbidities varied between municipalities and among the patient groups. This may explain a part of the spatial variation of treatment outcomes, since the percentage of multimorbid patients was found to correlate both with follow-up rates and with the management of risk factors. Individuals with multimorbidities had better follow-up attainment in all risk factors among male and female patients. At the municipality level, LDL-C treatment target attainment (<1.8 mmol/L) correlated with the proportion of comorbid patients (Figure 5). According to several studies, comorbid patients tend to have more primary care visits, even when confounders are taken into account [35], which could explain their better follow-up and attainment of targets rates. Due to multimorbidity, patients with a higher risk of a recurrent cardiac event may have better motivation for follow-up attainment and may have more frequent risk-factor screening and receive treatment that is more intensive.

### 4.6. Practical Usage of This Study

Results of this study helped to identify CHD patient groups with suboptimal treatment outcomes and areas with higher risk for CHD incidences and it revealed the differences in treatment outcomes, indicating similar differences to those found in research on spatial and SES differences among the outcomes of care of diabetic patients [36,37]. The study could be further developed with advanced geospatial analysis combined with individual level socio-economic characteristics and medical outcomes. If systematic flaws in secondary prevention among certain patient groups could be identified, then targeted approaches, like the Collaborative Model [38], could be used to improve guideline implementation. Although a study by Murphy et al. shows that the long-term secondary prevention risk factor management of coronary patients is challenging, as there were no clear improvements after 4–6 years of interventions, targeted cardiac secondary prevention programmes are beneficial for high-risk patients [12]. In addition to medical treatment, targeted and appropriate lifestyle interventions, including support for weight loss, dietary and physical activity counselling and smoking cessation should be more emphasized and targeted strategies developed for disadvantaged patient groups.

Improving both the thoroughness in recording activity and the coherence of patient records is crucial for productive assessment of quality of care. Developing the quality of patient records would help to improve auditing of secondary prevention and provide professionals with better access to quality reports on their patients. It could also help to improve the attendance to follow-ups by means of more efficient patient recall systems. The low recording activity may be, in part, explained by a suboptimal usability of patient information systems. More user-friendly ICT systems could improve the quality and coverage of the recordings [39].

### 4.7. Strengths and Limitations of This Study

A major strength of this study is that it relies on uniform and district-wide electronic patient records with geocodable information of patients’ domiciles, which made all these groupings and analyses possible. These records, which cover the entire research area, enabled the inclusion of practically all patients meeting the selected criteria and combining the cases with socio-economic and locational data. Furthermore, uniform laboratory test practices increase the reliability of data in this study.

The first limitation in the study is that data from private and occupational health-care are not fully included in the regional electronic patient records, and thus our data set omits some information about CHD patients who are in working life. These patients are nevertheless included in incidence rates, since all patients with ACS or invasive cardiac operations in the region are treated in public health care. The second limitation is that variation in recording activity and recording practices by primary care units affected the coverage and reliability of follow-up data. However, this variation in thoroughness of recording activity is an important practical finding of the study.

## 5. Conclusions

The study reveals that CHD incidences and CHD risk factors are strongly dependent on demographic and socio-economic factors leading to disparities between municipalities, settlement types and patient groups. Incident CHD cases and the severity of modifiable risk factors of CHD are associated more frequently with patients having a lower socio-economic status. The study region consists of one larger regional centre, small towns that are usually municipal centres and vast hinterlands. As expected, the population of the regional centre has the lowest probability of new CHD events whereas the small towns and remote areas have the highest risks for events. The spatial scan statistics analysis was used for detecting high- and low-risk areas of acute CHD. Low-risk areas were found within the commuter zone of the regional centre and the high-probability areas were in remote, mostly very sparsely populated rural areas. The results of incidence analysis at the municipal level support these findings. However, local deviations are found and should be included in further research; in particular, causes for the age-adjusted high-probability clusters should be studied. Both the socio-economic structure of the rural settlement type and the gender balance of the area may have influenced the probabilities of new CHD cases in these high-risk clusters. Geospatial analysis in the emergence of the incident CHD cases could be further developed by extending the study to include an analysis of risk factor management and by incorporating temporal variation into the analyses in order to study the quality of the health-care processes.

Demographic vulnerabilities in these types of regions, combined with unhealthy lifestyle factors usually related to low socio-economic status, accentuate morbidity disparities within regions. These long-evolved disparities are difficult to tackle [25,40]. At the municipality level, the sociodemographic status of the municipal population was strong but not an unambiguous explanatory factor for the uneven treatment outcomes. It is, however, clear that there is room for improvement in the prevention of recurrent cardiac events, as indicated by the low number of patients reaching guideline targets and the commonly low recording activity. Control of high blood pressure in particular was found to be in need of further attention as the proportion of recorded measurements varied substantially between areas and patient groups.

Attainment of BMI and blood pressure targets varied considerably between settlement types. This may indicate spatial characteristics of lifestyle factors aggravating the disparities beyond demographic differences. In addition, more patients being overweight and obese in areas of high incidence rates presumably indicates such differences in diet and other living habits that affect the other risk factors as well. Even though weight management in secondary prevention programmes has proven to be difficult [10], the attitudes and performance of professionals have also certainly influenced the differences in weight management outcomes between municipalities.

As attainment of treatment targets varied clearly between age, gender and settlement types, as expected, the variations in recording activity of most of the risk factors were larger between municipalities than between the above-mentioned classification, indicating organizational differences in recording and treatment practices. Hence, it is important to consider the effects of the possible differences in treatment practices in health centres in further analysis.

Multimorbidity, indicating a significantly elevated risk for recurrent events, was associated with better attainment of follow-up and treatment outcomes. However, regarding the long-term benefits, the appropriate treatment of patients with lower risk would be as important to avoid the development of multimorbid conditions. This would remarkably increase the quality of life of patients as well as decrease the health-care costs [41,42].

Overall, the variation in incidence rates, follow-up rates and attainment of treatment targets are notable. As demographic, socio-economic and lifestyle factors generate these disparities, improvements in primary prevention, risk factor management, and other treatment practices could mitigate these disparities. Moreover, very little is known about the possible barrier effects affecting accessibility of care, motivation of patients, and patient-professional relationships in the region.

The research showed that health-care processes could be enhanced by further implementation of more detailed electronic patient record data and by combining different databases. Apart from conventional data recording, using automated notifications from registers about missed follow-ups or alarming laboratory results could be implemented. In addition, utilizing patients’ own devices for follow-ups and self-measurements would be of great help in care, as well as being cost-efficient. Once patients’ registered data allow longer follow-up periods, these data should be more routinely used for analyses of treatment practices, quality outcome indicators, patient–professional communications and spatiotemporal analysis of treatment outcomes.

## Figures and Tables

**Table 1:** Performed coronary heart disease risk factor measurements and recordings of multimorbid and non-multimorbid patients with ACS or PCI/CABC in North Karelia Hospital District catchment area in 2011–2014.

	Multimorbid Patients						Patient with ACS or PCI/CABG only					
	Both Genders		Male		Female		Both Genders		Male		Female	
	*N*	%	*N*	%	*N*	%	*N*	%	*N*	%	*N*	%
Number of patients	1348	53%	845	51%	503	55%	1208	47%	800	49%	408	45%
Body mass index recorded *	873	65%	540	64%	333	66%	656	54%	438	55%	218	53%
HDL cholesterol recorded	992	74%	639	76%	353	70%	839	69%	566	71%	273	67%
LDL cholesterol recorded	1059	79%	667	79%	392	78%	896	74%	598	75%	298	73%
Triglycerides recorded	984	73%	638	76%	346	69%	819	68%	551	69%	268	66%
Blood pressure recorded	866	64%	517	61%	349	69%	558	46%	346	43%	212	52%
Smoking status recorded **	888	66%	541	64%	347	69%	539	45%	357	45%	182	45%

* Includes normal weight patients without recorded BMI measurement after cardiac event. Male *N* = 309, female *N* = 147; ** Includes non-smokers without recorded smoking status after cardiac event. Male *N* = 217, female *N* = 171; HDL = high-density lipoprotein, LDL = low-density lipoprotein, ACS = acute coronary syndrome, PCI = percutaneous coronary intervention, CABC = coronary artery bypass grafting.

## Supplementary Materials

The following are available online at http://www.mdpi.com/1660-4601/15/4/724/s1, Table S1: Measurements and recordings related to coronary heart disease (CHD) risk factor management classified by age, gender and settlement type in North Karelia Hospital District in 2011–2014; Table S2: Performed coronary heart disease (CHD) risk factor measurements and their levels by municipality classes in North Karelia Hospital District in 2011–2014; Table S3: Treatment outcomes of secondary prevention risk factor management among coronary heart disease (CHD) patients at age of 35–84 in North Karelia Hospital District in 2011–2014; Figure S1: Age-adjusted cumulative incidence rate of acute coronary heart disease and/or invasive treatment of male and female patients by municipalities in North Karelia Hospital District in 2011–2014.
