# Supplementary material for: Outcomes of Secondary Prevention among Coronary Heart Disease Patients in a High-Risk Region in Finland
Source: Int J Environ Res Public Health. 2018 Apr 11;15(4):724. doi: 10.3390/ijerph15040724 (PMC5923766; doi:10.3390/ijerph15040724)
Supplement: Supplementary file 1 [file ijerph-15-00724-s001.pdf]

Supplementary data

Table S1. Measurements and recordings related to coronary heart disease (CHD) risk factor management classified by age, gender and settlement type in North Karelia Hospital District in 2011–2014.

Gender	Age group	N	Settlement type	Smoking status		LDL cholesterol levels (mml/l)				Body mass index (BMI) levels				Blood pressure	
				**Smokers**	**Recorded**	**< 1.8**	**1.8<2.5**	**> 2.5**	**Recorded**	**< 25**	**25–29.9**	**> 30**	**Recorded**	**> 140/90**	**Recorded**
Both genders	35–64	297	Regional centre	*27 %*	*55 %*	*23 %*	*44 %*	*33 %*	*75 %*	*29 %*	*40 %*	*31 %*	*63 %*	*50 %*	*43 %*
	65–74	279		*8 %*	*62 %*	*28 %*	*42 %*	*30 %*	*78 %*	*32 %*	*34 %*	*35 %*	*61 %*	*60 %*	*54 %*
	75–84	262		*1 %*	*61 %*	*32 %*	*44 %*	*23 %*	*71 %*	*43 %*	*37 %*	*20 %*	*58 %*	*63 %*	*60 %*
Males	35–64	231		*26 %*	*55 %*	*24 %*	*46 %*	*30 %*	*75 %*	*22 %*	*40 %*	*31 %*	*62 %*	*51 %*	*41 %*
	65–74	162		*9 %*	*57 %*	*30 %*	*46 %*	*25 %*	*80 %*	*24 %*	*38 %*	*39 %*	*57 %*	*58 %*	*55 %*
	75–84	126		*1 %*	*62 %*	*37 %*	*47 %*	*16 %*	*75 %*	*43 %*	*45 %*	*12 %*	*59 %*	*58 %*	*57 %*
Females	35–64	66		*29 %*	*58 %*	*20 %*	*35 %*	*45 %*	*74 %*	*27 %*	*42 %*	*31 %*	*70 %*	*49 %*	*50 %*
	65–74	117		*7 %*	*69 %*	*25 %*	*36 %*	*39 %*	*75 %*	*38 %*	*31 %*	*31 %*	*67 %*	*61 %*	*53 %*
	75–84	136		*0 %*	*60 %*	*28 %*	*42 %*	*31 %*	*67 %*	*41 %*	*31 %*	*28 %*	*58 %*	*67 %*	*63 %*
Both genders	35–64	282	Rural centre	*28 %*	*62 %*	*29 %*	*36 %*	*36 %*	*81 %*	*25 %*	*35 %*	*40 %*	*70 %*	*46 %*	*55 %*
	65–74	339		*9 %*	*65 %*	*28 %*	*38 %*	*34 %*	*79 %*	*23 %*	*39 %*	*38 %*	*58 %*	*56 %*	*62 %*
	75–84	366		*3 %*	*60 %*	*29 %*	*41 %*	*30 %*	*77 %*	*35 %*	*37 %*	*28 %*	*55 %*	*48 %*	*67 %*
Males	35–64	211		*30 %*	*60 %*	*30 %*	*36 %*	*43 %*	*82 %*	*22 %*	*40 %*	*38 %*	*69 %*	*47 %*	*56 %*
	65–74	218		*10 %*	*66 %*	*33 %*	*38 %*	*37 %*	*77 %*	*21 %*	*44 %*	*35 %*	*58 %*	*54 %*	*60 %*
	75–84	169		*5 %*	*60 %*	*36 %*	*40 %*	*42 %*	*77 %*	*42 %*	*42 %*	*16 %*	*52 %*	*44 %*	*59 %*
Females	35–64	71		*21 %*	*68 %*	*23 %*	*34 %*	*43 %*	*79 %*	*34 %*	*22 %*	*44 %*	*72 %*	*43 %*	*52 %*
	65–74	121		*7 %*	*63 %*	*20 %*	*39 %*	*42 %*	*83 %*	*26 %*	*31 %*	*43 %*	*58 %*	*59 %*	*65 %*
	75–84	197		*2 %*	*60 %*	*22 %*	*42 %*	*36 %*	*76 %*	*30 %*	*33 %*	*38 %*	*57 %*	*50 %*	*74 %*
Both genders	35–64	268	Remote areas	*26 %*	*56 %*	*26 %*	*39 %*	*36 %*	*75 %*	*22 %*	*42 %*	*37 %*	*62 %*	*48 %*	*41 %*
	65–74	230		*6 %*	*63 %*	*35 %*	*34 %*	*31 %*	*81 %*	*24 %*	*41 %*	*35 %*	*59 %*	*55 %*	*55 %*
	75–84	224		*3 %*	*59 %*	*28 %*	*38 %*	*35 %*	*72 %*	*38 %*	*31 %*	*31 %*	*55 %*	*53 %*	*65 %*
Males	35–64	209		*28 %*	*56 %*	*26 %*	*40 %*	*34 %*	*75 %*	*21 %*	*42 %*	*37 %*	*63 %*	*44 %*	*42 %*
	65–74	173		*8 %*	*61 %*	*38 %*	*33 %*	*29 %*	*79 %*	*28 %*	*40 %*	*33 %*	*57 %*	*46 %*	*51 %*
	75–84	140		*3 %*	*55 %*	*31 %*	*40 %*	*30 %*	*74 %*	*41 %*	*29 %*	*30 %*	*56 %*	*49 %*	*61 %*
Females	35–64	59		*18 %*	*56 %*	*23 %*	*34 %*	*43 %*	*75 %*	*21 %*	*42 %*	*36 %*	*58 %*	*65 %*	*39 %*
	65–74	57		*3 %*	*68 %*	*27 %*	*37 %*	*37 %*	*86 %*	*16 %*	*43 %*	*41 %*	*65 %*	*76 %*	*65 %*
	75–84	84		*4 %*	*67 %*	*24 %*	*34 %*	*42 %*	*70 %*	*31 %*	*36 %*	*33 %*	*51 %*	*58 %*	*71 %*
		Standard deviation		*10.6%*	*4.2%*	*5.0%*	*4.1%*	*6.9%*	*4.2%*	*8.1%*	*5.5%*	*7.5%*	*5.5%*	*8.0%*	*9.3%*

Includes recordings after 30 days of diagnosis/procedure. Recordings of smoking status and BMI taken before that were also included when the person was a non-smoker / normal weight.

Table S2. Performed coronary heart disease (CHD) risk factor measurements and their levels by municipality classes in North Karelia Hospital District in 2011–2014.

Municipality		Smoking status		Low-density lipoprotein cholesterol (mmol/l)				Body mass index (BMI) levels				Blood pressure		Multimorbid patients
**Number**	**Socio-economic structure - classification**	**Smokers**	**Rec.**	**< 1.8**	**1.8<2.5**	**> 2.5**	**Rec.**	**< 25**	**25–29.9**	**> 30**	**Rec.**	**> 140/90**	**Rec.**	
1	A	*6 %*	*71 %*	*30 %*	*40 %*	*30 %*	*72 %*	*27 %*	*33 %*	*39 %*	*48 %*	*57 %*	*67 %*	*59 %*
2	A	*13 %*	*58 %*	*29 %*	*40 %*	*31 %*	*82 %*	*29 %*	*29 %*	*41 %*	*59 %*	*45 %*	*68 %*	*43 %*
3	B	*12 %*	*60 %*	*29 %*	*41 %*	*30 %*	*74 %*	*33 %*	*38 %*	*29 %*	*62 %*	*58 %*	*54 %*	*51 %*
4	A	*17 %*	*55 %*	*28 %*	*41 %*	*31 %*	*78 %*	*23 %*	*34 %*	*43 %*	*62 %*	*54 %*	*66 %*	*57 %*
5	B	*8 %*	*55 %*	*27 %*	*37 %*	*36 %*	*73 %*	*34 %*	*34 %*	*32 %*	*51 %*	*45 %*	*46 %*	*44 %*
6	C	*11 %*	*52 %*	*28 %*	*44 %*	*28 %*	*76 %*	*30 %*	*41 %*	*29 %*	*54 %*	*60 %*	*45 %*	*33 %*
7	A	*11 %*	*66 %*	*26 %*	*35 %*	*39 %*	*80 %*	*30 %*	*32 %*	*38 %*	*60 %*	*55 %*	*49 %*	*73 %*
8	C	*10 %*	*59 %*	*22 %*	*40 %*	*38 %*	*79 %*	*23 %*	*46 %*	*31 %*	*59 %*	*51 %*	*61 %*	*39 %*
9	B	*14 %*	*64 %*	*38 %*	*36 %*	*27 %*	*87 %*	*28 %*	*41 %*	*31 %*	*69 %*	*43 %*	*67 %*	*76 %*
10	B	*15 %*	*79 %*	*35 %*	*35 %*	*29 %*	*78 %*	*27 %*	*39 %*	*34 %*	*76 %*	*55 %*	*70 %*	*71 %*
11	A	*20 %*	*51 %*	*28 %*	*28 %*	*45 %*	*80 %*	*45 %*	*28 %*	*28 %*	*50 %*	*45 %*	*50 %*	*53 %*
12	A	*11 %*	*63 %*	*21 %*	*40 %*	*40 %*	*73 %*	*11 %*	*47 %*	*42 %*	*64 %*	*40 %*	*59 %*	*49 %*
13	A	*10 %*	*59 %*	*22 %*	*47 %*	*31 %*	*78 %*	*22 %*	*41 %*	*37 %*	*49 %*	*46 %*	*46 %*	*43 %*
14	A	*17 %*	*59 %*	*33 %*	*41 %*	*26 %*	*75 %*	*26 %*	*45 %*	*29 %*	*63 %*	*63 %*	*67 %*	*67 %*
**Females**		***8 %***	***63 %***	***23 %***	***38 %***	***39 %***	***76 %***	***32 %***	***33 %***	***35 %***	***61 %***	***58 %***	***62 %***	***55 %***
**Males**		***15 %***	***59 %***	***31 %***	***40 %***	***29 %***	***77 %***	***28 %***	***40 %***	***32 %***	***60 %***	***50 %***	***53 %***	***52 %***
**Both genders**		***12 %***	***60 %***	***28 %***	***40 %***	***32 %***	***77 %***	***30 %***	***38 %***	***33 %***	***60 %***	***53 %***	***56 %***	***53 %***
**Standard deviation of both genders**		***3.8 %***	***7.6%***	***4.8%***	***4.6%***	***5.6%***	***4.0%***	***7.7%***	***6.2%***	***5.4%***	***8.1%***	***7.1%***	***9.5%***	***13.5%***

Includes recordings after 30 days of diagnosis/procedure. Recordings of smoking status and BMI taken before that were also included when the person was a non-smoker / normal weight.

Municipalities' socio-economic structure classification:

A Sparsely populated, low education, unemployment, primary production, high demographic vulnerability

B Moderately dense population, high education, service

C High education, high employment, diverse industry, low demographic vulnerability.

Table S3. Treatment outcomes of secondary prevention risk factor management among coronary heart disease (CHD) patients at age of 35–84 in North Karelia Hospital District in 2011–2014.

**Male**			**Female**	
	**N**		**N**	
**Patients**	1645		911	
**BMI mean (95% CI)**	978	28.4 (28.1–28.7)	551	28.4 (27.8–28.9)
**LDL-C mean (95% CI)**	1265	2.2 (2.16–2.24)	690	2.4 (2.36–2.51)
**Triglycerides mean (95% CI)**	1189	1.38 (1.33–1.43)	614	1.32 (1.27–1.37)
**Triglycerides < 1.7**	932	78.4% (76–80.7%)	492	80.1% (77–83.3%)
**HDL mean (95% CI)**	1205	1.3 (1.23–1.27)	626	1.5 (1.49–1.55)
**HDL (<1.0/1.2)**	307	25.5% (23–27.9%)	143	22.8% (19.6–26.1%)
**HDL (>1.0/1.2)**	898	74.5% (72.1–77%)	483	77.2% (73.9–80.4%)
**non-HDL mean (95% CI)**	1196	2.60 (2.55–2.65)	621	2.79 (2.70–2.87)
**Systolic blood pressure (mean)**	856	140.9 (139.5–142.3)	558	146.1 (144.5–147.7)
**Systolic blood pressure (<140)**	430	49.4% (45.7–53.1%)	236	43% (38.7–47.4%)
**Diastolic blood pressure (mean)**	863	79.5 (78.4–80.6)	561	79.1 (78.3–80)
**Diastolic blood pressure (<90)**	576	82.2% (79.3–85%)	413	82.3% (78.9–85.6%)

BMI=Body mass index, HDL=high-density lipoprotein, LDL=low-density lipoprotein, CI=confidence interval.

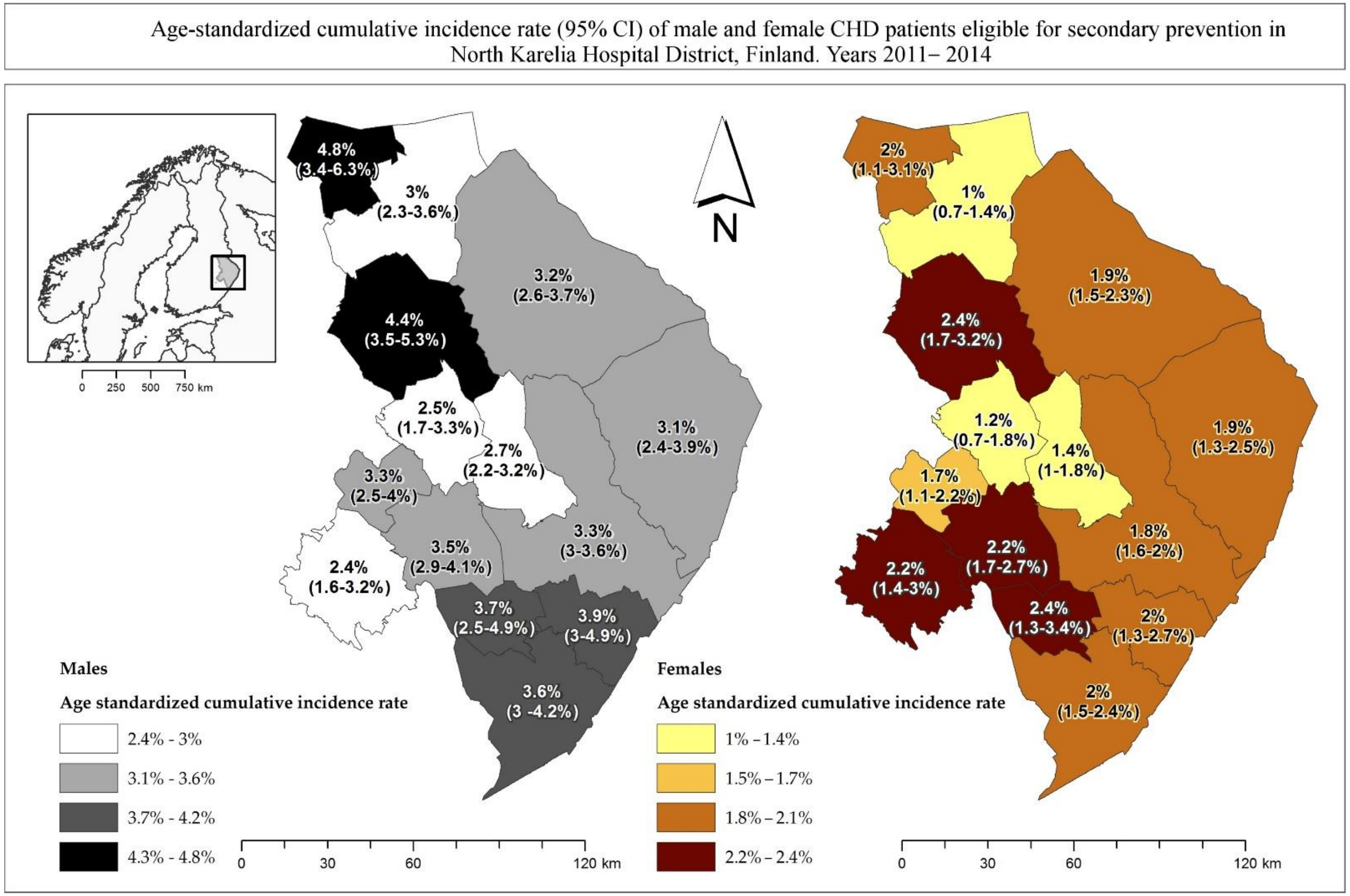

Figure S1. Age-adjusted cumulative incidence rate of acute coronary heart disease and/or invasive treatment of male and female patients by municipalities in North Karelia Hospital District in 2011–2014.
